# *Sporidiobolus pararoseus* wall-broken powder ameliorates oxidative stress in diabetic nephropathy in type-2 diabetic mice by activating the Nrf2/ARE pathway

**DOI:** 10.1039/c8ra10484k

**Published:** 2019-03-14

**Authors:** Yuliang Cheng, Chang Liu, Yan Cui, Tianqi Lv, Yahui Guo, Jun Liang, He Qian

**Affiliations:** School of Food Science and Technology, Jiangnan University 1800 Lihu Avenue Wuxi Jiangsu Province P. R. China amtf168168@126.com; Synergetic Innovation Center for Food Safety and Nutrition, Jiangnan University Wuxi 214122 China; School of Food Science, State Key Laboratory of Food Science and Technology, National Engineering Research Center for Functional Foods, Jiangnan University Wuxi 214122 China; Institute of Agricultural Products Processing, Key Laboratory of Preservation Engineering of Agricultural Products, Ningbo Academy of Agricultural Sciences Ningbo 315040 China; Guangzhou GRE Metrology & Test Co., Ltd Guangzhou Guangdong 510000 PR China

## Abstract

In type 2 diabetes mellitus (T2DM), hyperglycemia promotes oxidative stress and eventually leads to diabetic nephropathy (DN). *Sporidiobolus pararoseus* is reported to exhibit enhanced anti-oxidation properties. However, its role in DN remains obscure. This study aimed to determine the antioxidative effects of a *Sporidiobolus pararoseus* wall-broken powder (SPP) supplement on DN and investigate the possible underlying mechanisms. A model of T2DM was successfully established, and C57BL/6J male mice were fed a high-fat diet for 4 weeks and then injected with streptozotocin (100 mg per kg per day) for three consecutive days. After eight weeks of intervention, SPP strongly lowered fasting glucose levels, serum creatinine, serum urea nitrogen, urinary albumin and reduced glomerular hypertrophy and mesangial matrix expansion. In addition, SPP increased the activities of SOD, T-AOC, CAT, and GST and decreased the amount of MDA. Furthermore, it was revealed that SPP significantly abrogated oxidative stress not only by activating the Nrf2 gene but also by activating two Nrf2-targeted antioxidative genes (NQO-1 and HO-1) compared with metformin hydrochloride, which is widely accepted as a diabetes drug. Our study showed that SPP has antioxidant properties and delays the progression of DN; the underlying mechanism may be associated with activation of the Nrf2/ARE pathway.

## Abstract 2

STZ-induced diabetic mice are given a high-fat diet and SPP, which is a rich source of β-carotene, γ-carotene, torulene and torularhodin. The result indicated SPP can ameliorate diabetic nephropathy *via* activating Nrf2/ARE pathway.

## Graphical abstract

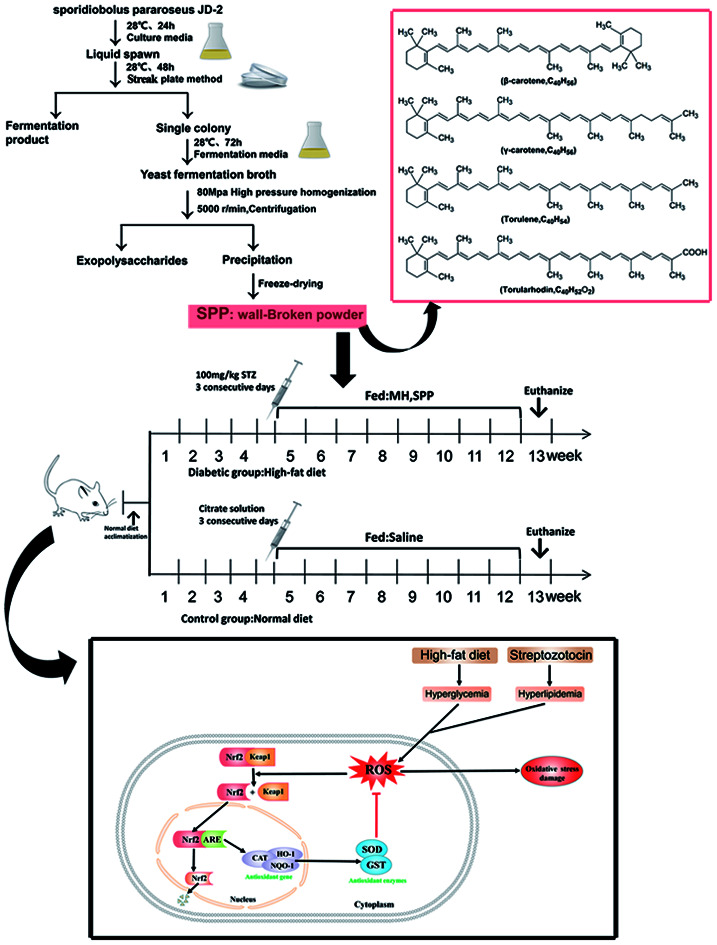

## 1. Introduction

Diabetes mellitus (DM) is one of the most common metabolic diseases worldwide, and it occurs due to either insulin resistance or insufficient levels of insulin.^1^ It is concerning that type 2 diabetes (T2DM) is caused by obesity; cases of T2DM are rapidly increasing and may result in a worldwide epidemic.^2,3^ Furthermore, hyperglycemia in diabetes ultimately leads to long-term damage and complications, including heart and cardiovascular diseases, tissue damage to the liver and pancreas, diabetic ophthalmopathy and chronic kidney disease.^4^ Previous studies demonstrated that the morphological characteristics of diabetic nephropathy (DN) include glomerular hypertrophy, tubular atrophy of the basement, mesangial expansion, membrane thickening, interstitial fibrosis and arteriolar thickening.^5^ Diabetes can lead to different degrees of pathological renal damage, and renal hemodynamics alters glomerular permeability.^6^ Persistent hyperglycemia increases the risk of diabetic nephropathy characterized by renal failure; as a result, DN is becoming one of most prominent long-term diabetic complications and is a major source of morbidity and mortality.^7–9^

Generally, oxidative damage plays an important role in the pathogenesis of DN.^10^ Oxidative stress can be easily caused by disproportion of reactive oxygen species (ROS) and endogenous antioxidants. A proper level of ROS helps the body to survive and proliferate; however, high levels of ROS can lead to apoptosis and damage of cellular macromolecules, including DNA, proteins and lipids.^11–13^ Symptoms of diabetic nephropathy include hyperglycemia and poor glucose control. Hyperglycemia stimulates oxidative stress in the body, and a series of protective proteins are induced in order to eliminate excessive ROS to protect the body and its cells from oxidative damage.^14^ The antioxidant response element is the main regulator of this defense system. Additionally, nuclear erythroid 2-related factor 2 (Nrf2) plays a key regulatory role in the mechanism of antioxidant stress. The DNA promoter sequence, antioxidant responsive element (ARE), is often found at the 5′ end of superoxide dismutase (SOD), glutathione S-transferase (GST), catalase (CAT) and other genes. Oxidative stress can activate ARE and enhance the expression of two Nrf2-targeted antioxidant genes, namely NAD(P)H quinone oxidoreductase 1 (NQO-1) and heme oxygenase-1 (HO-1), and antioxidant enzymes such as SOD and CAT.^15,16^ The Nrf2/ARE signaling pathway is crucial for endogenous antioxidant stress, which is of great significance in alleviating and treating DN. Therefore, antioxidation may effectively protect normal renal function and delay the progress of DN.^17^

*Sporidiobolus pararoseus* is a facultative aerobic yeast belonging to the class Basidiomycetes; it has strong adaptability and wide distribution in nature. Its colony color is usually red or light red. *Sporidiobolus pararoseus* has been widely used in the food industry because it can produce many nutrients, such as pigments, unsaturated fatty acids and polysaccharides.^18^ In our previous study, the red yeast *Sporidiobolus pararoseus* JD-2 was isolated from pepper sauce (save number: CCTCC M2010326).^19^ This yeast has the ability to accumulate high microbial oils and also can produce extracellular polysaccharides, fatty acids, ergosterol, squalene and carotenoids.^19–21^ According to our previous study, extract of the red yeast *Sporidiobolus pararoseus* JD-2 is rich in carotenoids; also, four main kinds of carotenes were identified in SPP by HPLC, namely β-carotene, γ-carotene, torulene and torularhodin.^21,22^ Studies have showed that the extracts of *Sporidiobolus pararoseus* have the ability to ameliorate hyperlipidemia induced by a high-fat diet in obese mice as well as protect human prostate stromal cells from oxidative stress induced by hydrogen peroxide.^23,24^ Therefore, *Sporidiobolus pararoseus* extract has a strong antioxidant effect and may have positive effects on DN; however, this regulation effect has not yet been reported.

Currently, many drugs are used to treat diabetic nephropathy; however, these drugs have many side effects compared with other natural products.^25^ Although natural plants and herbs with medicinal functions have been used in the treatment of diabetic nephropathy, there are still many difficulties to overcome because of their complex extraction and purification processes.^26^ Therefore, it is necessary to identify foods with minimal side effects and simple extraction methods to treat diabetic nephropathy. This study investigated the effects of a wall-broken powder supplement of *Sporidiobolus pararoseus* on ameliorating diabetic nephropathy.

## 2. Materials and methods

### 2.1 Materials and chemicals

*Sporidiobolus pararoseus* JD-2 (preservation number: CCTCC M2010326) was obtained and characterized by our laboratory; STZ and all fine chemicals were obtained from Sigma (St Louis, MO, USA); metformin hydrochloride (MH) was purchased from Bangde Pharmaceutical (Shandong province, China); commercial assay kits for superoxide dismutase (SOD), total antioxidant capacity (T-AOC), malondialdehyde (MDA), creatinine and urea nitrogen were purchased from Nanjing Jiancheng Technology Co. (Nanjing, China); antibodies against Nrf2, HO-1, NQO1 and GST were purchased from Santa Cruz Biotechnology (Dallas, TX, United States). All other chemicals were purchased from local companies and were of analytical grade.

### 2.2 Preparation of *Sporidiobolus pararoseus* wall-broken powder (SPP)

*Sporidiobolus pararoseus* JD-2 was stored at −80 °C. The yeast was inoculated in a conical flask containing culture media and incubated at 28 °C for 24 h with horizontal rocking. Yeast in liquid media that exhibited coloration were cultured in Petri dishes at 28 °C for approximately 48 h. The pigmented colonies were then transferred to fermentation media and incubated at 28 °C for 72 h. All experiments were performed in an environment with constant humidity. Yeast in fermentation media were homogenized using 80 MPa and centrifuged for 10 min at 5000 rpm. SPP was obtained by freeze-drying from precipitation and stored at −4 °C.

### 2.3 Animals

Forty-eight eight-week-old male C57BL/6J mice [SPF, SCXK (Hu) 2013001823759] (20 ± 2 g) were obtained from SiLaiKe Laboratory Animal Company (Shanghai, China). Mice were housed in environmentally standard cages at a constant temperature (22 ± 1 °C) and humidity (a relative humidity of 60 ± 10%). The mice were exposed to a 12 : 12 h light–dark cycle-altered room with food and water available. Mouse procedures and protocols were carried out according to the Institutional Animal Care and Use Committee of Jiangnan University, Wuxi, China [Approval no. JN no. 20170328-20170829(37)].

### 2.4 Experimental design

Mice were fed a normal diet to acclimate for 7 days and then given a high-fat diet, excluding the control group mice, which were given a normal diet. The high-fat diet was composed of 1.5% cholesterol, 0.2% sodium cholate, 5% sugar, 15% lard oil, 0.7% salt, 0.8% additive trace element, and 76.8% of a commercial standard pellet diet. After 4 weeks, STZ (100 mg per kg per day) dissolved in fresh sodium citrate buffer (pH 4.5, 0.1 mol L^−1^) was injected intraperitoneally into the mice for 3 consecutive days.^27^ Accordingly, mice in the control group were given equivalent volumes of fresh sodium citrate buffer. Three days after STZ induction, blood samples were drawn from the tail veins of mice that were fasted overnight. Mice with blood glucose levels above 11.1 mmol L^−1^ were considered diabetic. The mice were randomly divided into six groups (*n* = eight). SPP and MH were dissolved in 0.9% saline and then fed to the mice for 8 weeks by gavage daily. The volume of gavage was 40 mL kg^−1^.

Control group: normal diet + 0.9% saline

Model group: high-fat diet + 0.9% saline

MH group: high-fat diet + 1.5 mg kg^−1^

SPP-L group: high-fat diet + 1.5 g^−1^ kg^−1^

SPP-M group: high-fat diet + 3.0 g^−1^ kg^−1^

SPP-H group: high-fat diet + 6.0 g^−1^ kg^−1^

### 2.5 Body weight, feed intake, water intake, fasting blood glucose and organ index

The mice were weighed on the last day of every week. Daily food intake, daily water intake and fasting blood glucose were determined on the same day. After 8 weeks of administration, the mice were anesthetized and then sacrificed. Blood samples from all mice that were fasted overnight were collected. Blood plasma was collected through the retro-orbital venous plexus, followed by centrifugation at 5000 rpm at 4 °C for 10 min; the serum was then stored at −80 °C for further analyses. After the mice were sacrificed, the heart, liver, kidney, and spleen were removed and accurately weighed. The visceral index was calculated as organ weight (g)/body weight (kg). Kidney tissues were excised and fixed in normal 4% paraformaldehyde for histopathological evaluation, while other kidney tissues were maintained at −80 °C for further experiments.

### 2.6 24 hour urine volume, urinary albumin, serum creatinine and serum urea nitrogen

The mice were placed into metabolic cages for 24 h on the same day of every week. Urine was collected at the end of the experiment. The levels of urinary albumin, serum creatinine and serum urea nitrogen were analyzed *via* biochemical kits from Nanjing jiancheng Technology Co. (Nanjing, China). Briefly, serum creatinine was reacted with different enzyme solutions at 37 °C; then, the absorbency was measured at a wavelength of 546 nm. The final results were obtained by comparing with the standard. Serum urea nitrogen was mainly reacted with enzyme solution and chromogenic agent at 37 °C; then, the absorbance was measured at a wavelength of 640 nm, and the final value was obtained by comparing the test results with the standard sample.

### 2.7 Serum MDA, SOD and T-AOC

The levels of SOD, T-AOC and MDA in the serum of control and diabetic mice were analyzed using commercial assay kits obtained from Nanjing Jiancheng Technology Co. (Nanjing, China) according to the manufacturer's instructions. In the MDA test, the homogenate and working liquid were mixed and placed in a 95 °C water bath for 40 minutes. The supernatant was obtained by centrifuging and measured at an absorbance of 530 nm, and the final results were obtained. In the SOD test, the homogenate, working liquid and enzyme solution were mixed at 37 °C for 20 minutes; then, the absorbance was measured at a wavelength of 450 nm, and the final value was obtained by comparing the test results with the standard sample and blank sample.

### 2.8 Kidney MDA, SOD and T-AOC

After sacrifice, kidneys were collected, and one part of each kidney was homogenized in 0.9% saline with three washes in ice-cold physiological saline. After being centrifuged at 12 000 rpm for 15 min at 4 °C, the homogenate was collected in tubes and stored at −80 °C. The protein contents were analyzed with a BCA protein assay kit from Nanjing Jiancheng Technology Co. (Nanjing, China). The levels of T-AOC, SOD and MDA in the kidney tissues were analyzed using commercial kits purchased from Nanjing Jiancheng Technology Co. (Nanjing, China). All procedures were carried out according to the manufacturers' instructions.

### 2.9 Histopathological examination

The kidney samples (*n* = 8) that were excised at the end of the experiment were fixed in normal 4% paraformaldehyde in sodium phosphate buffer for 48 h. The fixed tissues were then dehydrated in a graded alcohol series, cleared with xylene, and embedded in paraffin wax. The tissues were then sliced into 5 μm-thick sections and stained with hematoxylin and eosin (H&E); periodic acid-Schiff (PAS) staining was performed according to the standard protocol. The pathological alterations in the kidneys were then assessed under light microscopy with magnifications of 200× and 400×. Histological examinations were performed using an inverted microscope (Zeiss, Germany).

### 2.10 Immunohistochemical examination

After deparaffinization and hydration to activate endogenous antigens, the 5 μm-thick slides were washed in tris-buffered saline (TBS). Slides were quenched by a methanol solution containing 3% H_2_O_2_. Subsequently, the slides were washed with PBS 3 times. The slices were incubated with 10% rabbit serum at 37 °C for 60 min. Incubation with primary antibodies (anti-Nrf2 antibody (SC-722) and anti-NQO1 antibody (SC-32793), Santa Cruz Biotechnology, USA) was performed at 4 °C overnight. The slides were then washed in TBS three times. The tissue sections were incubated with secondary antibodies (goat anti-rabbit (SC-2091), goat anti-mouse (SC-362257), Santa Cruz Biotechnology, USA) at 37 °C for 60 min. The slides were then washed with PBS 3 times. The time of color development was controlled under the light microscope. Slides were stained with hematoxylin, dehydrated with different gradients of alcohol and then sealed. The images were captured by an inverted microscope (Zeiss, Germany).

### 2.11 RNA extraction and quantitative real-time PCR

In brief, we extracted the total RNA from the kidney tissues with an RNA isolation kit (Shanghai Generay Biotech Co., Ltd) according to the manufacturer's instructions. cDNA was generated from 1 μg of total RNA and was reverse transcribed according to standard protocols from the strand synthesis system in the RT-PCR kit (Shanghai Generay Biotech Co., Ltd). The other steps of transcription and extension by the PCR reaction were carried out as described previously.^24^ The designed primer sequences are listed in Table 1.

**Table 1:** Sequences of primers for quantitative real-time PCR

Gene	Sequence 5′–3′ (forward)	Sequence 5′–3′ (reverse)
β-Actin	GGGAAATCGTGCGTGAC	AGGCTGGAAAAGAGCCT
HO-1	GAGTGGGGCATAGACTGGGTT	GCTGGTGATGGCTTCCTTGTA
GST	GCGTTCCACCTTCTCGTCAGT	CGCATTCCAGAGGATAACCAA
CAT	GGTCGGTCTTGTAATGGAACTT	CACATGAATGGCTATGGATCA
Nrf2	GAAAAAGAAGTGGGCAACTGTGG	GGTGGGATTTGAGTCTAAGGAGGT
NQO1	GGTATTACGATCCTCCCTCAACATC	GAGTACCTCCCATCCTCTCTTCTTC

### 2.12 Statistical analyses

GraphPad Prism 7 was used for statistical analysis. The analysis results are expressed as the mean ± standard error of the mean (SEM). All the data obtained in this study were analyzed using one-way analysis of variance (ANOVA) followed by Tukey's *y* test. *P* < 0.05 was considered significant.

## 3. Results

### 3.1 Influence on body weight, food intake, water intake and fasting blood glucose

At the beginning of the experiment, the body weights of the mice in each group increased steadily, without significant difference. However, after intraperitoneal injection of STZ, the body weight decreased while the daily feed intake, drinking water intake and fasting blood glucose level increased in mice in the diabetic group, which suggests that STZ successfully induces diabetes.

**Fig. 1:**
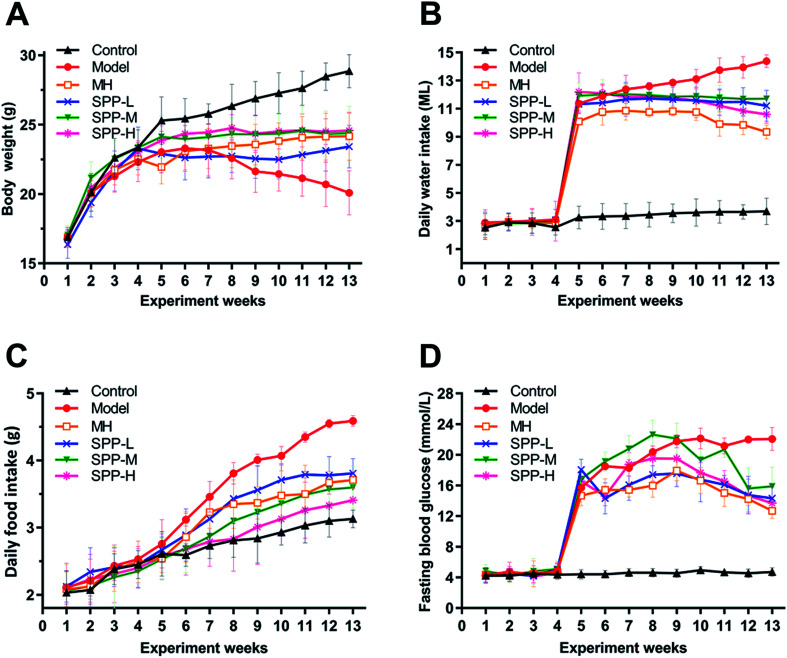
Effects of *S. pararoseus* JD-2 wall-broken powder on body weight (A), daily water intake (B), daily food intake (C) and fasting blood glucose (D) in diabetic nephropathy mice.

As indicated in (Fig. 1A), body weight was significantly decreased in the diabetic mice compared to the nondiabetic control mice. In contrast, the diabetic mice showed marked increases in elevated daily water intake, daily food intake and fasting blood glucose levels (Fig. 1B–D). After MH and SPP extract administrations, food intake, water intake and fasting blood glucose levels were significantly reduced. No significant differences in body weight, daily food intake, daily water intake and fasting blood glucose levels were observed between the MH group and SPP group.

### 3.2 Influence on organ index

The body weight loss and increase of the organ index are considered as the typical diabetic characteristics induced by STZ. The organ indices of the heart, liver and kidneys in the diabetic group exhibited notable increases compared to those of the control group (Table 2). The index for the kidneys also showed a significant increase in the diabetic group when compared with the control group (*P* < 0.01). However, there was no obvious change in the spleen tissues of the different MH and SPP groups.

**Table 2:** Effects of *S. pararoseus* wall-broken powder on organ indices in DN mice^a^

Organ	Organ indices (g kg^−1^)					
	Control	Model	MH	SPP-L	SPP-M	SPP-H
Heart	6.25 ± 0.73	4.57 ± 0.97^#^	5.15 ± 0.25	5.47 ± 0.21	5.09 ± 0.80	5.47 ± 0.56
Liver	42.56 ± 4.09	65.11 ± 2.64^#^	62.09 ± 3.28	64.10 ± 3.52	67.16 ± 4.34	60.41 ± 3.75
Kidney	7.68 ± 1.65	17.93 ± 0.88^##^	11.75 ± 0.45*	11.21 ± 1.00**	13.25 ± 0.73*	11.11 ± 0.87**
Spleen	2.98 ± 0.61	3.57 ± 0.93	3.25 ± 0.53	3.17 ± 0.40	2.85 ± 0.66	3.42 ± 0.62

a Values are expressed as the mean ± SEM of 8 mice per group. Model group *vs.* control group: ^#^*p* < 0.05, ^##^*p* < 0.01. MH group, SPP group *vs.* Model group: *p < 0.05, **p < 0.01.

### 3.3 Influence on 24 hour urine volume and urinary albumin

**Fig. 2:**
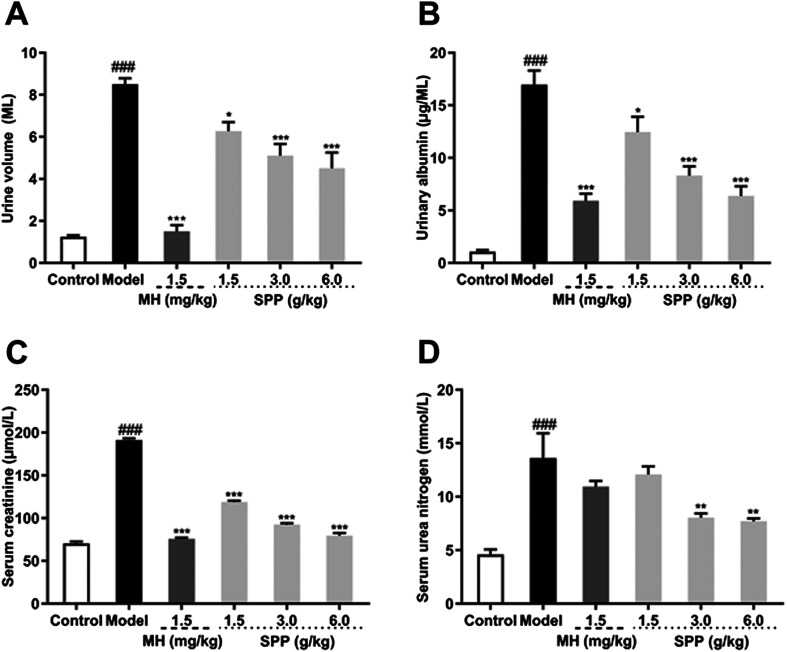
Effects of *S. pararoseus* JD-2 wall-broken powder on urine volume for 24 h after 8 weeks of treatment (A), urinary albumin after 8 weeks of treatment (B), serum creatinine (C), and serum urea nitrogen (D) in diabetic nephropathy mice. Values are expressed as the mean ± SEM of 8 mice per group. Model group *vs.* control group: ^###^*p* < 0.001. MH group, SPP group *vs.* Model group: *p < 0.05, **p < 0.01, ***p < 0.001.

Urine volume and urinary albumin were increased in STZ-induced diabetic mice compared with mice in the control group (*P* < 0.001). Additionally, the administration of MH and SPP ameliorated urinary albumin and urine volume after 24 h. After MH and SPP extract administration, urine volume and urinary albumin were significantly decreased, especially after treatment with SPP (3 g kg ^−1^ and 6 g kg ^−1^) (Fig. 2A and B). Further, it was observed that SPP can significantly decrease the 24 hour urine volume and urinary albumin in mice, recover renal damage and prevent the occurrence and development of diabetic nephropathy.

### 3.4 Influence on serum creatinine and serum urea nitrogen

Results from the biochemical plasma study revealed that STZ significantly (*P* < 0.001) impaired kidney function as assessed by an increase in serum creatinine and serum urea nitrogen levels when compared to the control group (Fig. 2C and D). Significant differences were observed in mice that were administered SPP, and levels were decreased when compared with the diabetic group. SPP also ameliorated the decreased creatinine levels. The calculated serum urea nitrogen clearance of the diabetic group was markedly higher when compared to the SPP group (3 g kg^−1^ and 6 g kg^−1^).

### 3.5 Influence on MDA, SOD and T-AOC of serum

**Fig. 3:**
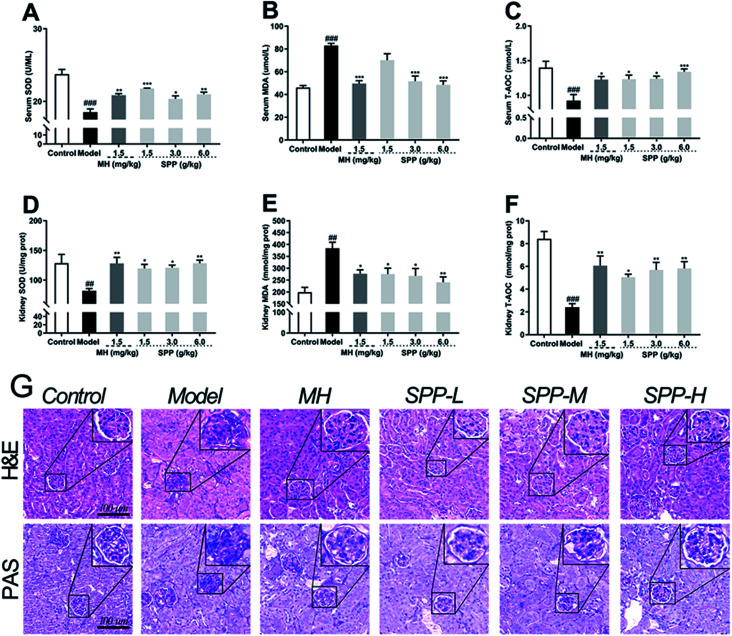
Effects of *S. pararoseus* JD-2 wall-broken powder on serum SOD (A), serum MDA (B), serum T-AOC (C), kidney SOD (D), kidney MDA (E) and kidney T-AOC (F) in diabetic nephropathy mice. Effects of *S. pararoseus* JD-2 wall-broken powder on kidney histoarchitecture of high-fat diet and STZ-induced early diabetic nephropathy in mice. Kidney sections were stained with periodic acid-Schiff staining (H&E, PAS) (G) (original magnification, ×400) and hematoxylin and eosin (original magnification, ×400). Values are expressed as the mean ± SEM of 8 mice per group. Model group *vs.* control group: ^##^*p* < 0.01, ^###^*p* < 0.001. MH group, SPP group *vs.* Model group: *p < 0.05, **p < 0.01, ***p < 0.001.

The oxidative stress that occurred in kidney cells was investigated. The results showed that diabetic mice had significant decreases in the bioactivities of SOD and T-AOC in the kidneys (*P* < 0.001); in turn, the MDA level was elevated (Fig. 3B, *P* < 0.001). Compared to STZ-induced diabetic mice, MH and SPP treatment resulted in a significant decrease in serum MDA levels and significant increases in serum T-AOC and SOD activities (Fig. 3A and C). However, there was no significant difference in the SOD activity of the SPP (1.5 g kg^−1^) group when compared with that of the diabetic group.

### 3.6 Influence on MDA, SOD and T-AOC levels of kidney

Lipid peroxidation was determined by measuring the levels of MDA, T-AOC and SOD. As shown in Fig. 3, STZ stimulation significantly decreased the activities of SOD and T-AOC (*P* < 0.001). The SPP (1.5 g kg^−1^ and 3 g kg^−1^) treatments and MH treatments effectively restored the levels of SOD and T-AOC (*P* < 0.05), which were weaker than those in the SPP (6.0 g kg^−1^) group. In addition, a high level of MDA was found in the diabetic group (Fig. 3E, *P* < 0.01). In contrast, the administration of SPP (1.5 g kg^−1^ and 3 g kg^−1^) and SPP (6.0 g kg^−1^) dramatically decreased the MDA levels (*P* < 0.01).

### 3.7 Histopathological analysis

Analysis of tissues from control mice showed normal glomerular and tubular structures (Fig. 3G). The diabetic group showed degenerated glomeruli, mesangial expansion, and thickened basement membranes because there were increases in collagen accumulation in the MH and SPP groups compared with the control group. SPP significantly ameliorated the incidence of glomerular basement membrane thickening and mesangial proliferation as well as inflammatory infiltrate injuries in the kidneys of diabetic mice.

We examined glycogen accumulation in the kidneys by periodic acid-Schiff (PAS) staining, which showed that diabetes induced significant accumulation of glycogen. This effect was significantly prevented by SPP treatment. PAS-positive areas indicating an increased number of glycogen-filled proximal tubules and extracellular matrix deposits were all decreased by SPP (6.0 g kg^−1^) administration.

### 3.8 Immunohistochemical analysis

**Fig. 4:**
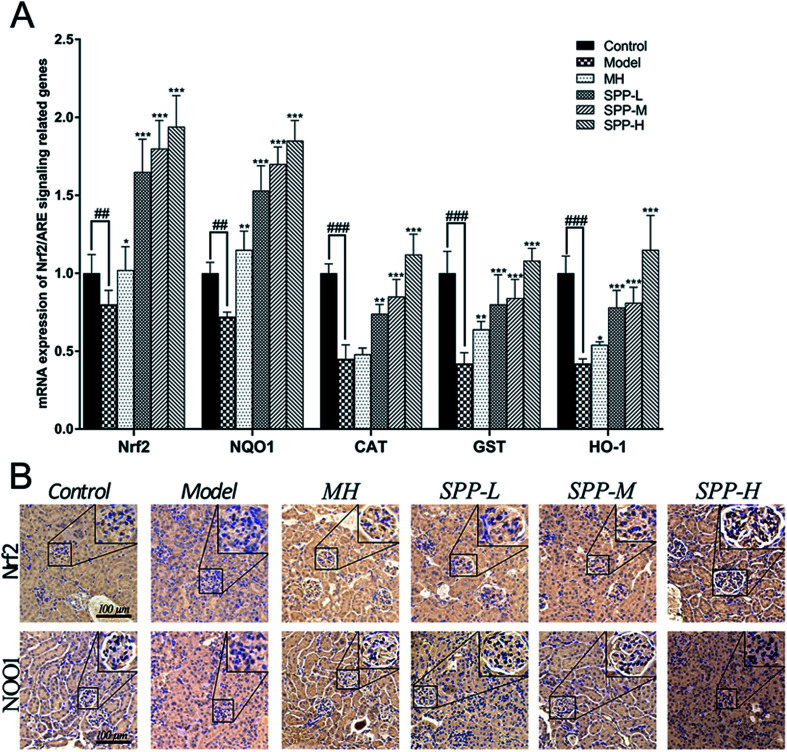
Effects of *S. pararoseus* JD-2 wall-broken powder on the mRNA expression of proteins in the kidney. The mRNA levels of Nrf2, NQO1, CAT, GST and HO-1 were determined by qPCR (A). Effects of *S. pararoseus* JD-2 wall-broken powder on kidney immunohistochemical staining of high-fat diet and STZ-induced early diabetic nephropathy in mice. Immunohistochemical staining for Nrf2 and NQO1 with brown color (original magnification, ×400) (B). Values are expressed as the mean ± SEM of 8 mice per group. Model group *vs.* control group: ^##^*p* < 0.01, ^###^*p* < 0.001. MH group, SPP group *vs.* Model group: *p < 0.05, **p < 0.01, ***p < 0.001.

Immunohistochemical staining for Nrf2 and NQO1 was assayed in the kidney tissue samples (Fig. 4B). Diabetic mice showed decreased expression of Nrf2 in the intraglomerular area when compared with the control kidney samples. SPP decreased renal Nrf2 expression in diabetic mice. Immunohistochemical analysis of the kidney tissue sections revealed that NQO1 expression in the glomeruli was visibly decreased in the diabetic mice compared to that in the control group. Increased renal NQO1 accumulation was markedly decreased by MH and SPP treatment. Moreover, these results indicated that the diabetes-induced oxidation formation and accumulation in the kidneys were dramatically controlled by SPP.

### 3.9 mRNA expression levels of Nrf2-related antioxidant genes

The results indicate concomitant decreases (*P* < 0.01) in the mRNA expression levels of Nrf2 and NQO1 in kidneys derived from STZ-treated mice when compared to the control; the expression levels of Nrf2 and NQO1 were downregulated by approximately 1.4 fold (Fig. 4A). Importantly, gene expression was significantly (*P* < 0.001) downregulated, as shown in the mRNA expression levels of CAT, GST and HO-1 in the kidneys of the diabetic groups compared with the control group; the expression levels of CAT, GST and HO-1 were downregulated by more than twofold in the kidneys of the diabetic groups in comparison to the control group. The administration of MH alone did not show significant effects when compared with the diabetic group.

After SPP administration for 8 weeks, intrarenal mRNA expression levels were elevated in a dose-dependent manner (*P* < 0.01). Compared with the diabetic control, the increases in the mRNA expression levels of Nrf2, NQO1, CAT, GST and HO-1 were inhibited by SPP treatment to different degrees. The middle and high doses of SPP significantly restored the expression levels of Nrf2, NQO1, CAT, GST and HO-1 (*P* < 0.001); especially, Nrf2, NQO1, and HO-1 expression were significantly upregulated by more than twofold.

## 4. Discussion

This study is the first to demonstrate the protective effects of *Sporidiobolus pararoseus* on DN in an experimental model of T2DM. Previous studies confirmed that carotenoids in serum can reduce the risk of T2DM, which can be achieved by supplementing the diet with fruits and vegetables. Lycopene has therapeutic effects on DN induced by STZ in mice *via* attenuating oxidative stress for anti-dysmetabolism.^28^ According to our previous study, extract of the red yeast *Sporidiobolus pararoseus* JD-2 is rich in carotenoids; there are four main kinds of carotene in the SPP, namely β-carotene, γ-carotene, torulene and torularhodin.^21,22^ Therefore, we assumed that SPP attenuates the injury of DN in T2DM.

Previous studies have shown that a high-fat diet can induce insulin resistance mainly through the fatty acid cycling of glucose.^29,30^ As a result of the excessive intake of fat, triglyceride metabolism increases, eventually causing an increase in fatty acid content, resulting in oxidative damage. When an increased amount of fatty acid is consumed as quickly as possible, insulin-mediated inhibition of hepatic glucose output can be attenuated, and glucose uptake is decreased, which may result in diabetes.^31^ ROS may mediate a possible increase in free fatty acids. In addition, STZ-induced diabetes is characterized by ROS damage to pancreatic beta cells, leading to insufficient insulin synthesis, which contributes to the development and progression of diabetes and its complications such as DN.^32,33^ Many studies have reported that the C57BL/6J strain is an effective model to study the absence of glomerular mesangial expansion in the kidney tissue of diabetic mice, and it was observed that PAS-positive staining of kidneys is significantly increased.^34,35^

It is known that the body weight loss, feed intake and the increase in the organ index are considered to be the classical characteristics in T2DM induced by a high-fat diet and STZ administration.^36,37^ The increased kidney index in DN indicates the development of renal hypertrophy.^38^ The results indicate that SPP can ameliorate the decrease in body weight and kidney index seen in diabetic mice, and SPP treatment showed a significant effect on daily food and water intake. As a result of STZ administration, blood glucose levels in the mice remained greater than 11.1 mmol L^−1^. In parallel, SPP administration effectively prevented the increase in blood glucose, which further demonstrates that SPP promoted glycemic bioavailability and decreased pathological damage in DN. Hyperglycaemia in diabetes results in the production of reactive oxygen species (ROS) and cell death.^39^ A possible mechanism is that SPP effectively scavenges the free radicals produced by oxidative stress *in vivo* and protects islet cells from free radicals so that the islet cells can normally produce insulin and inhibit the increase of blood glucose.

Hyperglycemia weakens the glomerular filtration barrier, causes glomerular damage, and leads to albumin leakage, which exacerbates DN.^40^ Scr and SUN levels in renal lesions or dysfunctions were generally elevated, suggesting that hyperglycemia can lead to renal damage.^41^ Consistent with those interpretations, our study showed decreases in Scr, SUN, urine volume and albuminuria. However, oral treatment with SPP significantly decreased these levels, suggesting that SPP effectively ameliorates diabetic renal injury induced by hyperglycemia.

Enhancement of oxidative stress is one of the most basic causes of chronic complications in T2DM.^42^ Oxidative stress refers to the imbalance between oxidative damage and antioxidant capacity *in vivo*; this is more likely to produce a large number of oxidative intermediates, resulting in aggravated oxidative damage *in vivo*. When the organism is stimulated by various harmful substances, excessive ROS and reactive nitrogen free radicals (RNS) are produced. The antioxidant capacity is weakened as a result of excessive ROS.^43^ The changes in oxidative stress markers such as MDA and SOD indicate oxidative stress *in vivo*.^44^ Studies have shown that MDA increases and SOD decreases in STZ-induced DN.^45,46^ SOD is the first barrier to free radical oxidative damage, which is to remove free radicals.^47^ In addition, MDA is the end-product of lipid peroxidation, indicating the degree of oxidative damage.^48^ The antioxidant capacity of T-AOC directly reflects the ability of the body to remove free radicals.^49^ Thus, our study indicates that these oxidative stress markers demonstrate the antioxidative potential of SPP treatment in a high-fat diet and STZ-induced diabetic mice. When antioxidant function was further impaired in diabetic mice, SPP treatment significantly decreased MDA levels and replenished the levels of T-AOC and SOD towards normal levels in both the serum and kidney.

The theories of Nrf2/ARE pathway activation in DN have been confirmed over the past ten years.^50^ To our knowledge, Nrf2 is considered to be the primary cellular defense against oxidative stress.^51^ The Nrf2/Keap1 (kelch-like epichlorohydrin-related protein 1) system regulates the transcription of antioxidant and cytoprotective genes through direct binding of Nrf2 to responsive elements in the promoter regions of target genes.^52^ In normal cells, Nrf2 is inhibited by the inhibitor Keap 1 and is found in the cytoplasm. To accumulate in the nucleus, Nrf2 is detached from Keap 1 and activates the expression of ARE-driven genes such as HO-1 and NQO1.^53,54^ In addition, upregulation of GST was found to occur *via* an antioxidant regulation mechanism in response to oxidative stress.^55^ Therefore, the activation of Nrf2 may be a method to prevent or slow the progression of DN. Consistent with these results, high glucose levels induced mRNA expression levels of HO-1, NQO1, and GST in diabetic mice.

## 5. Conclusion

Carotenoids from a wide range of sources are abundant in nature, have low toxicity, and can be produced in large quantities in the metabolic pathways of *Sporidiobolus pararoseus* JD-2. The results of this study provide a theoretical basis for development of the carotenoids in *Sporidiobolus pararoseus* JD-2 as a natural antidiabetic product. Based on our results, we can speculate that DN is connected to an increase in oxidative stress and that SPP treatment can inhibit DN *via* activating the Nrf2 signaling pathway.

## Conflicts of interest

The authors declare that they have no conflict of interest.

## Abbreviations

SPP: *Sporidiobolus pararoseus* wall-broken powder
DM: Diabetes mellitus
DN: Diabetic nephropathy
STZ: Streptozotocin
T2DM: Type 2 diabetes mellitus
Nrf2: Nuclear erythroid 2-related factor 2
ARE: Antioxidant response element
NQO1: NAD(P)H quinone oxidoreductase 1
HO-1: Heme oxygenase-1
Keap 1: Kelch-like epichlorohydrin-related protein 1
ROS: Reactive oxygen species
GST: Glutathione S-transferase
H&E: Hematoxylin and eosin
PAS: Periodic acid-Schiff
MH: Metformin hydrochloride
RNS: Reactive nitrogen free radicals
SOD: Superoxide dismutase
T-AOC: Total antioxidant capacity
MDA: Malondialdehyde
CAT: Catalase

## Supplementary Material

## References

[cit1] Ryden A., Sorstadius E., Bergenheim K., Romanovschi A., Thoren F., Witt E. A., Sternhufvud C. (2016). PLoS One.

[cit2] Namekawa J., Takagi Y., Wakabayashi K., Nakamura Y., Watanabe A., Nagakubo D., Shirai M., Asai F. (2017). J. Vet. Med. Sci..

[cit3] Heydemann A. (2016). J. Diabetes Res..

[cit4] Chen Z., Wang C., Pan Y., Gao X., Chen H. (2018). Food Funct..

[cit5] Kashihara N., Haruna Y., Kondeti V. K., Kanwar Y. S. (2010). Curr. Med. Chem..

[cit6] Liu Y., Bledsoe G., Hagiwara M., Yang Z. R., Shen B., Chao L., Chao J. (2010). Am. J. Physiol. Renal. Physiol..

[cit7] Singh D. K., Winocour P., Farrington K. (2011). Nat. Rev. Endocrinol..

[cit8] Gnudi L. (2012). Nephrol., Dial., Transplant..

[cit9] Kanwar Y. S., Wada J., Sun L., Xie P., Wallner E. I., Chen S., Chugh S., Danesh F. R. (2008). Exp. Biol. Med..

[cit10] Nguyen T., Nioi P., Pickett C. B. (2009). J. Biol. Chem..

[cit11] Siednienko J., Gorczyca W. A. (2003). Postepy Hig. Med. Dosw..

[cit12] Chen X., Wei S. Y., Li J. S., Zhang Q. F., Wang Y. X., Zhao S. L., Yu J., Wang C., Qin Y., Wei Q. J., Lv G. X., Li B. (2016). PLoS One.

[cit13] Zheng H., Whitman S. A., Wu W., Wondrak G. T., Wong P. K., Fang D., Zhang D. D. (2011). Diabetes.

[cit14] Zhao C. R., Gao Z. H., Qu X. J. (2010). Cancer Epidemiol..

[cit15] Cui W., Min X., Xu X., Du B., Luo P. (2017). J. Diabetes Res..

[cit16] Shin D. H., Park H. M., Jung K. A., Choi H. G., Kim J. A., Kim D. D., Kim S. G., Kang K. W., Ku S. K., Kensler T. W., Kwak M. K. (2010). Free Radical Biol. Med..

[cit17] Chorley B. N., Campbell M. R., Wang X. T., Karaca M., Sambandan D., Bangura F., Xue P., Pi J. B., Kleeberger S. R., Bell D. A. (2012). Nucleic Acids Res..

[cit18] Buzzini P., Innocenti M., Turchetti B., Libkind D., van Broock M., Mulinacci N. (2007). Can. J. Microbiol..

[cit19] Han M., He Q., Zhang W. G. (2012). Prep. Biochem. Biotechnol..

[cit20] Han M., Xu Z. Y., Du C., Qian H., Zhang W. G. (2016). Bioprocess Biosyst. Eng..

[cit21] Shi Q. Y., Wang H. Y., Du C., Zhang W. G., Qian H. (2013). Anal. Sci..

[cit22] Han M., He Q., Zhang W. G. (2012). Prep. Biochem. Biotechnol..

[cit23] Du C., Ying D., Guo Y., Cheng Y., Han M., Zhang W., Qian H. (2018). Biochem. Cell Biol..

[cit24] Du C., Guo Y., Cheng Y., Han M., Zhang W., Qian H. (2017). Free Radical Res..

[cit25] Ravindran S., Kuruvilla V., Wilbur K., Munusamy S. (2017). J. Cell. Physiol..

[cit26] Bilal M., Iqbal M. S., Shah S. B., Rasheed T., Iqbal H. M. N. (2018). Recent Pat. Inflammation Allergy Drug Discovery.

[cit27] Ishak N. A., Ismail M., Hamid M., Ahmad Z., Abd Ghafar S. A. (2013). Evid.-Based Complementary Altern. Med..

[cit28] Guo Y., Liu Y. H., Wang Y. X. (2015). Food Funct..

[cit29] Randle P. J., Garland P. B., Hales C. N., Newsholme E. A. (1963). Lancet.

[cit30] Kraegen E. W., Clark P. W., Jenkins A. B., Daley E. A., Chisholm D. J., Storlien L. H. (1991). Diabetes.

[cit31] Rosholt M. N., King P. A., Horton E. S. (1994). Am. J. Physiol..

[cit32] Bandeira S. D., da Fonseca L. J. S., Guedes G. D., Rabelo L. A., Goulart M. O. F., Vasconcelos S. M. L. (2013). Int. J. Mol. Sci..

[cit33] Nishikawa T., Araki E. (2007). Antioxid. Redox Signaling.

[cit34] Schlondorff D. (2010). Kidney Int..

[cit35] Shao Y. X., Xu X. X., Wang K., Qi X. M., Wu Y. G. (2017). Drug Des., Dev. Ther..

[cit36] Sezik E., Aslan M., Yesilada E., Ito S. (2005). Life Sci..

[cit37] Badal S. S., Danesh F. R. (2014). Am. J. Kidney Dis..

[cit38] Pal P. B., Sinha K., Sil P. C. (2014). PLoS One.

[cit39] Ji L., Liu Y., Zhang Y., Chang W., Gong J., Wei S., Li X., Qin L. (2016). Can. J. Physiol. Pharmacol..

[cit40] Ma S. T., Liu D. L., Deng J. J., Niu R., Liu R. B. (2013). Phytother. Res..

[cit41] Yanardag R., Bolkent S., Ozsoy-Sacan O., Karabulut-Bulan O. (2002). Phytother. Res..

[cit42] Wu Y., Tang L., Chen B. (2014). Oxid. Med. Cell. Longevity.

[cit43] Bondeva T., Wolf G. (2014). Nephrol., Dial., Transplant..

[cit44] Anjaneyulu M., Chopra K. (2004). Pharmacology.

[cit45] Sharma S., Kulkarni S. K., Chopra K. (2006). Clin. Exp. Pharmacol. Physiol..

[cit46] West I. C. (2000). Diabetic Med..

[cit47] Kawamoto S., Inoue M., Tashiro S., Morino Y., Miyauchi Y. (1989). Transplant. Proc..

[cit48] Donate-Correa J., Martin-Nunez E., Muros-de-Fuentes M., Mora-Fernandez C., Navarro-Gonzalez J. F. (2015). J. Diabetes Res..

[cit49] Shao N., Kuang H. Y., Wang N., Gao X. Y., Hao M., Zou W., Yin H. Q. (2013). J. Diabetes Res..

[cit50] Miyata T., Suzuki N., de Strihou C. V. (2013). Kidney Int..

[cit51] Jimenez R., Toral M., Gomez-Guzman M., Romero M., Sanchez M., Mahmoud A. M., Duarte J. (2018). Oxid. Med. Cell. Longevity.

[cit52] Zoja C., Benigni A., Remuzzi G. (2014). Nephrol., Dial., Transplant..

[cit53] Shelton L. M., Park B. K., Copple I. M. (2013). Kidney Int..

[cit54] Kobayashi M., Yamamoto M. (2005). Antioxid. Redox Signaling.

[cit55] Chan K., Han X. D., Kan Y. W. (2001). Proc. Natl. Acad. Sci. U. S. A..

